# Development of the autoinflammatory disease damage index (ADDI)

**DOI:** 10.1186/1546-0096-13-S1-P29

**Published:** 2015-09-28

**Authors:** K Annink, N ter Haar, M Gattorno, H Lachmann, J Kuemmerle-Deschner, S Ozen, R Goldbach-Mansky, K Durrant, O Della Casa Alberighi, J Frenkel

**Affiliations:** 1 University Medical Center Utrecht, Utrecht, Netherlands; 2 G. Gaslini Institute, Genoa, Italy; 3 University College London Medical School, London, UK; 4 University Hospital Tuebingen, Tuebingen, Germany; 5 Hacettepe University Faculty of Medicine, Ankara, Turkey; 6 National Institute of Health, Bethesda, USA; 7 Autoinflammatory Alliance, San Francisco, USA

## Introduction

Autoinflammatory diseases cause systemic inflammation which can result in damage to multiple organs. Organ damage can occur before the start of therapy, or when patients experience ongoing inflammation. A validated instrument to measure damage is essential to quantify damage in individual patients, and to compare disease outcomes in clinical studies. At this moment, there is no such instrument. In the context of the RaDiCEA project, a common damage index for Familial Mediterranean Fever (FMF), Cryopyrin Associated Periodic Syndromes (CAPS), Tumor Necrosis Factor Receptor Associated Periodic Syndrome (TRAPS) and Mevalonate Kinase Deficiency (MKD) will be developed.

## Objectives

To develop an autoinflammatory disease damage index (ADDI).

## Methods

The ADDI will be developed by consensus building based on the Delphi method. The top 40 enrollers of autoinflammatory disease patients in the Eurofever registry, were invited to take part in the expert group, as well as nine important experts from the Americas. A systematic literature search for damage in autoinflammatory diseases was performed to establish possible damage items. These items were rated by the experts in an online survey. Experts could also provide new damage items that were not found in the literature. Furthermore, in close collaboration with the Autoinflammatory Alliance, twenty-two patients and parents of patients were asked to rate damage items from the literature and to suggest other damage items. Based on the comments and suggestions of experts and patients, the damage items will be adapted; multiple rounds of online surveys will be performed to reach consensus. The final damage index will be determined in a face-to-face consensus meeting.

## Results

Fifty-seven possible damage items from the literature were selected for the online survey. Forty of forty-nine experts completed the first round of the online survey. Based on the first round, five items were excluded and seventeen new items were suggested by experts. Twenty-one patients completed the online survey. Patients suggested nineteen new items, for example learning disabilities, chronic fatigue and speech development delay. The second round will be adapted based on these results.

## Conclusion

An instrument to measure damage caused by autoinflammatory diseases (ADDI) will be developed based on the Delphi consensus procedure, including online surveys and a consensus meeting. Patients fulfil a significant role in this process.

